# Patient characteristics in a return to work programme for common mental disorders: a cross-sectional study

**DOI:** 10.1186/s12889-016-3431-0

**Published:** 2016-08-08

**Authors:** Mattias Victor, Bjørn Lau, Torleif Ruud

**Affiliations:** 1 Lovisenberg Hospital, Oslo, Norway; 2 Department of Psychology, University of Oslo, Oslo, Norway; 3 Division Mental Health Services, Akershus University Hospital, Lørenskog, Norway; 4 Institute of Clinical Medicine, University of Oslo, Oslo, Norway

**Keywords:** Return to work, Common mental disorders, Psychological treatment, Sick-leave, Sickness absence, Sick-leave benefits, Mental health, Psychiatry, Outpatients, Norway

## Abstract

**Background:**

Mental health problems are a growing cause of sickness absence. There are programmes in many countries to facilitate return to work (RTW) after sickness absence. In Norway, there has been some controversy about patients on sick-leave being prioritized over other patient groups, such as those with more severe diagnoses. However, it is not clear whether patients in RTW programmes actually do differ from patients in regular services.

**Methods:**

This study compared 270 patients treated in an RTW outpatient clinic and 86 patients treated in a regular outpatient clinic, both in specialized mental health care, on patient characteristics, history of treatment and mental health status. Analyses of differences between groups were done by ANOVA tests, chi-square test and logistic regression.

**Results:**

Patients in the RTW clinic had lower scores on the Clinical Outcomes in Routine Evaluation Outcome Measure (CORE-OM). There was no difference in health-related quality of life. RTW patients were somewhat older and more likely to live in relationships and have children, and they had higher incomes. Work participation, previous psychiatric hospitalization and present diagnosis contributed uniquely to an explanation of which patients were included in the respective clinics. The RTW clinic seems to reach its intended target group. Almost all of the patients in this group participated in the work arena, and their psychopathologies were clearly dominated by common mental disorders. Most RTW patients’ general practitioners had followed them fairly closely in the year before referral, suggesting previous attempts at treatment in primary care settings.

**Conclusions:**

Relative to outpatients in a specialized mental health care setting, RTW patients had lower symptoms, but still in the same moderate range of severity. They suffered the same reduction in quality of life. Almost all of the RTW patients were diagnosed with illnesses that can be treated effectively, about half of them had recurring mental health problems and many of them had been treated in primary care settings before referral. These findings indicate that this group has significant health problems that can benefit from treatment in specialized health care settings.

## Background

Mental health problems are a growing cause of sickness absence [1, 2], and in recent years they have become a major occupational health issue in many countries [3, 4]. The prevalence of mental disorders peaks during working age [1], and therefore a large part of the workforce is affected [5]. These disorders negatively impact occupational functioning [6], and affected employees lose three times more work days a year than do other employees [7].

In Norway, mental disorders are the second most common reason for sickness absences overall [8], and the most common cause of long-term sickness absences. In 2013, mental disorders accounted for 18.3 % of the sickness absences documented on doctors’ certificates [8]. However, because mental disorders are often under-reported as a cause of sick-leave, it is likely that the actual percentage is higher [9]. In the Netherlands, mental disorders account for 30 % of all sickness absences longer than 1 year, an increase from 11 % in the late 1960s [2]. In a British study, mild mental disorders accounted for nearly 40 % of all sickness absences certified by general practitioners (GPs) [10]. In addition, mental disorders are associated with an increased risk of disability pension claims [11–15]. In the Organisation for Economic Co-operation and Development (OECD), mental disorders account for one-third of all new disability pension claims, on average, and up to 40–50 % in some member states [16]. This share has almost doubled in the past 10–15 years in some countries.

Mental disorders can be divided into two subgroups: common mental disorders (CMDs) and severe mental disorders [17]. CMDs, especially depression and anxiety, contribute the most to the economic burden of reduced workdays [18]. These disorders affect more people than do severe mental disorders [19], and many people with CMDs are working [20]. Severe mental disorders (e.g. schizophrenia and other psychoses) are rarer, and people afflicted with them are less likely to be in paid employment [6]. There are effective psychological treatments for most CMDs, particularly depression and anxiety [21]. Treatment is less costly than financing benefit payments [22]. Nevertheless many people never get effective treatment [23], mainly because secondary mental health services focus on severe mental illness. Treatment of CMDs is usually provided by primary care providers, where the detection rate is low and treatment often sub-optimal [24].

Many countries have interventions to facilitate and hasten employees’ return to work (RTW) after sickness absence [25], and some of these interventions focus on mental health problems [26–31]. These interventions include such treatments as cognitive behavioural therapy, graded activity and workplace adaptations [25]. Norway initiated a national RTW programme in 2007 that includes a range of interventions for both somatic and mental health care problems [32]. The main goal of this programme is to reduce the cost to society of sick-leave benefits. By expanding treatment capacity, it was hoped that people on sick-leave would spend less time on waiting lists and return to work more quickly. To be included in the programme, patients must have a job and be entitled to sick-leave benefits. (In Norway, a person is entitled to sick-leave benefits after working for at least 4 weeks, and can receive sick-leave benefits for a maximum of 52 weeks; after that, patients can apply for other types of benefits.) Patients are referred by their GPs, and can either be on sick-leave already or be considered (by their GPs) to be at risk of requiring sick-leave. Thus, the programme also has a preventive function. Interventions in mental health care are supposed to focus on “less severe mental disorders”, usually interpreted as CMDs. The programme is said to follow a “bottom-up” approach, implying that local health care providers are free to design interventions without specific instructions from the authorities [32]. Consequently, local programmes vary in the interventions they offer. Interventions do not necessarily have a specific focus on rehabilitation. In Britain, the Improving Access to Psychological Therapies programme (IAPT) has largely expanded capacity for psychological treatment for CMDs in recent years [21, 33]. The focus on reducing waiting lists by expanding the overall treatment capacity implies that the Norwegian programme perhaps has more in common with IAPT than with more specific RTW programmes. In a sense, the Norwegian program can be viewed as an IAPT for people in employment.

The Norwegian RTW programme is an extraordinary intervention within the healthcare system, financed directly over the state budget and with a specially defined target group. There has been some ethical controversy in Norway about patients on sick-leave being prioritized over other patient groups, possibly including those with more severe diagnoses [34, 35]. As far as we know, no one has studied whether patients in the Norwegian RTW programme differ from patients in regular services, nor has anyone examined whether the RTW programme actually reaches its target group. A better understanding of this could inform future health care policies on RTW interventions.

In this paper, we analyse data on patients who received mental health care treatment through an RTW programme and those who received treatment in a regular outpatient clinic at a community mental health centre. We address the following questions: (a) Do the patients treated in the RTW programme differ significantly from the patients treated in the regular mental health outpatient clinic, when it comes to symptom severity, health-related quality of life, history of psychiatric treatment and background characteristics? (b) Which patient characteristics help to differentiate patients in the RTW programme from those who used the regular mental health outpatient clinic? (c) Does the RTW clinic reach the target group for the programme; i.e. patients with CMDs and a present work participation, entitled to sick-leave benefits? Based on the inclusion criteria for the RTW programme, our hypotheses were that patients in the RTW programme would have better mental health at the beginning of treatment, a history of previous treatment indicating less severe problems, and a greater likelihood of work participation. We expected the two groups to have similar socio-demographic variables.

## Methods

### Design

The study compares patients from a regular psychiatric outpatient clinic and patients from an RTW outpatient clinic for people on sick-leave or at risk of requiring sick-leave because of mental health problems. Both services are part of the Lovisenberg Community Mental Health Centre in Oslo. The treatment offered in the RTW clinic is individual, short-term psychotherapy and/or psycho-educative courses for various problems such as depression, social phobia, panic disorder, stress or sleep problems.

### Recruitment and sample

Participants were recruited during ten consecutive months (August 16^th^, 2010, to June 15^th^, 2011) from patients who attended their first session at either the RTW clinic or the regular outpatient clinic. All new patients were eligible to participate in this study; 573 patients in the RTW clinic and 267 patients in the regular outpatient clinic. One hundred and seventy-three (30 %) of the eligible patients at the RTW clinic and 117 (44 %) of the eligible patients at the regular outpatient clinic were not asked to participate, primarily because the therapists forgot to ask the patients to participate or because the therapists misunderstood which patients should be asked.

### Procedures

Fifty-seven therapists participated in the study, 41 (27 female) in the RTW clinic and 16 (13 female) in the regular outpatient clinic. Mean age of therapists were 39 years in the RTW clinic and 44 years in the regular outpatient clinic. In the RTW clinic 40 therapists were clinical psychologists and one was a doctor specialized in occupational medicine. In the regular outpatient clinic 12 therapists were clinical psychologists, two were psychiatrists and two were doctors specializing in psychiatry.

In the first session, patients received an information statement and were also verbally informed about the study by the therapist. If patients gave their written consent to participate, they were asked to fill in a paper-and-pencil questionnaire covering socio-demographics, work situation, mental health and quality of life. The completed questionnaires were either handed directly to the therapist or returned to the clinic by mail. In the next session, the therapists asked if the patient had returned the questionnaire and, if necessary, reminded the patients two more times in the following sessions. The therapists diagnosed the patients, and filled in a form with questions covering each patient’s present problem and treatment history. Patient questionnaires and therapist forms were first linked by the patient’s name and date of birth. Before patient questionnaires and therapist forms were filed, the patients name and date of birth were removed and replaced by a unique code known only to the researcher.

### Measurements

#### Patient characteristics

Socio-demographic information was collected on age, gender, number of children, marital status, educational level and income. Respondents were asked to indicate their level of work participation. Considering various combinations, a new variable was computed with six exclusive categories: 1) Fully working; 2) Partially working (respondents who worked part time and were either on sick-leave, receiving a social benefit or partially unemployed); 3) Full sick-leave; 4) No participation in the labour market (primarily unemployed or receiving other forms of social benefits that do not involve participation in the labour market, or combinations thereof); 5) School/studies (respondents who were in school/studying full time, including those working additional hours part time or receiving some form of social benefit); and 6) Other (respondents who did not fit into any of the other categories, such as being on maternity leave). The first three categories also indicate that respondents were entitled to sick-leave benefits. Some of the students in category 5 might also have been entitled to sick-leave benefits, depending on how much they worked parallel to their studies. We used a dichotomized version of this variable in the logistic regression analyses. The first three response categories were combined to indicate work participation, and the three latter categories were combined to indicate lack of work participation. To assess the use of health care services, patients were asked how many times they had visited a GP in the past year and how many times they had been admitted to hospital in the past 3 years. The therapists reported each patient’s previous mental health treatment history and current psychopharmacological medications.

#### Mental health status

Both self-administered and therapist-administered measures were used to assess mental health. Symptoms were measured with the self-administered Clinical Outcomes in Routine Evaluation Outcome Measure (CORE-OM) [36–38]. The CORE-OM consists of 34 items related to the previous week that address four domains: Problems (depression, anxiety, physical problems, trauma); Functioning (general day-to-day functioning, close relationships, social relationships); Subjective well-being (feelings about self and optimism about the future); and Risk (risk to self, risk to others). All items are scored from 0 (“never”) to 4 (“almost all the time”). Eight forms with less than 90 % of the items completed were excluded from analyses of total score. Mean scores are usually multiplied by 10 before being presented as a total score ranging from 0 to 40 [39]. Split scores for the four domains can also be calculated in the same way. A total score of 10 has been suggested as a clinical cut-off [40]. Total scores can also be used to indicate six levels of severity: Healthy (0–5), Low (6–9), Mild (10–14), Moderate (15–19), Moderate–Severe (20–24) and Severe (>25) [41]. Evans et al. have reported an internal consistency for the CORE-OM of Cronbach’s coefficient (α) = 0.94 and a 1-week test–retest reliability of Spearman’s *r* = 0.90 [38]. In line with Evans et al., we found a Cronbach’s coefficient (α) = 0.93 in this study.

Health-related quality of life (HRQoL) was measured with the 15D. This is a generic, standardized, self-administered instrument that adheres conceptually to the World Health Organization’s (WHO) definition of health as being composed of physical, mental and social well-being [42]. The 15D has been used to describe HRQoL for a broad range of clinical and general populations, including those with mental disorders [43]. The questionnaire consists of 15 items with a five-point scale for each that ranges from normal functioning (1) to severe problems (5). A set of preference weights elicited from the general public is used to generate a profile and a 15D score on a scale from 1 (no problems on any dimension) to 0 (lowest possible quality of life). A difference of ≥0.015 in the 15D score is considered clinically important [44]. The reliability, validity, sensitivity, discriminatory power and responsiveness to change of the 15D are comparable with other generic HRQoL instruments, such as the EQ-5D and the SF-6D [42, 45, 46].

The Global Assessment of Functioning (GAF) is a 100-point scale from the DSM-IV (Diagnostic and Statistical Manual of Mental Disorders – Fourth Edition) that is used to make a global rating of a patient’s social, occupational and psychological functioning (1 is the lowest score and 100 is the highest) [47]. Since 1998, Norwegian clinicians have used a split version the GAF, with one scale for symptoms (GAF-S) and one scale for social functioning (GAF-F).

The therapists diagnosed the patients according to ICD-10 guidelines, and the ICD-10 diagnoses were clustered into four categories: 1) Common mental disorders (including a depressive episode (F32), recurrent depressive episodes (F33), dysthymia (F34.1), phobic anxiety disorders (F40), other anxiety disorders (F41), obsessive-compulsive disorder (F42) and reaction to severe stress and adjustment disorders (F43)); 2) Severe mental disorders (including psychosis (F20-F29), a manic episode (F30) and bipolar disorder (F31)); 3) Other psychiatric diagnoses (e.g. substance abuse or eating disorder); and 4) Z-diagnoses (reasons for contact with health services not resulting in a psychiatric diagnoses, e.g. examination).

### Analyses: statistical methods

Data were analysed using SPSS for Windows (version 22). Differences between groups were analysed using ANOVA tests for continuous and ordinal variables and chi-square tests for categorical variables. Univariable and multivariable logistic regression analyses were performed to examine differences between the two patient groups. For the multivariable logistic regression analysis, the independent variables that were significant in the univariable analyses were entered simultaneously as predictors of the dependent variable.

## Results

### Subjects

**Fig. 1:**
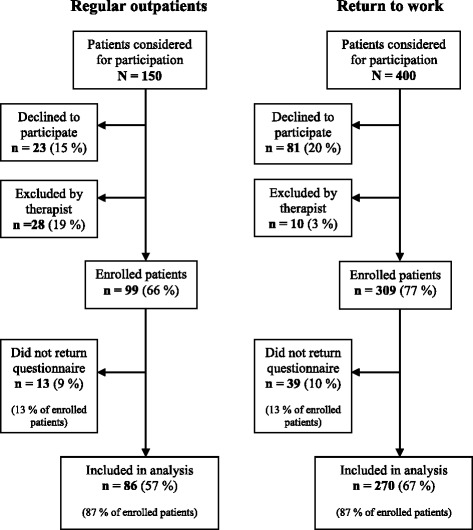
Flowchart of patient recruitment. Flowchart of patient recruitment, with percentages for subgroups of all patients considered for participation and for subgroups of included patients

Figure 1 shows the outcome for the 550 (65 %) patients who were considered for participation in this study. The therapists excluded 38 of these patients, mainly because participation would have been a burden for the patients or because the patients did not know the Norwegian language well enough to complete the questionnaire. Of the 408 patients who consented to participate, 52 did not return the questionnaire. The final set of completed questionnaires represented data from 356 patients, including 270 from the RTW clinic and 86 from the regular outpatient clinic.

To investigate potential selection bias, we examined whether there were differences between those who returned the questionnaire and all other eligible patients, for each clinic separately. Data was collected from medical records for all patients in the population. No statistically significant differences were found for age, gender, GAF scores or diagnoses. Therefore, we concluded that the sample did not differ significantly from the population on any of these variables, for any of the two clinics.

### Patient characteristics

As shown in Table 1, analyses of socio-demographics showed significant differences on all of the variables, except gender and education. Patients in the RTW clinic were older (M = 37.9, SD = 10.0, compared to M = 33.6, SD = 11.6; *p* = 0.001), were more likely to have children (*p* = 0.003), had higher incomes (*p* = 0.000) and were more likely to live with a partner (*p* = 0.003).

**Table 1:** Patient characteristics

		Regular outpatients *N* = 86	Return to work *N* = 270	Significance testing Chi-square (sig.)
		*N* (%)	*N* (%)	
Socio-demographics				
Age (years)	18–29	38 (44.2 %)	53 (19.6 %)	23.065 (0.000)***
	30–39	31 (36.0 %)	119 (44.1 %)	
	40–49	7 (8.1 %)	57 (21.1 %)	
	50–	10 (11.6 %)	41 (15.2 %)	
Gender	Men	36 (41.9 %)	83 (30.7 %)	3.624 (0.057)
	Women	50 (58.1 %)	187 (69.3 %)	
Marital status	Living alone	58 (69.0 %)	136 (50.6 %)	8.841 (0.003)**
	Living with partner	26 (31.0 %)	133 (49.4 %)	
Children	Yes	13 (15.3 %)	86 (32.0 %)	8.916 (0.003)**
Education	Comprehensive school (1–9 years)	6 (7.0 %)	15 (5.6 %)	2.354 (0.502)
	Secondary/vocational school (10–12 years)	27 (31.4 %)	80 (29.7 %)	
	College degree (13–16 years)	43 (50.0 %)	124 (46.1 %)	
	Higher university degree (>16 years)	10 (11.6 %)	50 (18.6 %)	
Income in NOK	Under 200,000	49 (57.0 %)	32 (12.2 %)	76.124 (0.000)***
	200,000–299,000	13 (15.1 %)	45 (17.2 %)	
	300,000–399,000	13 (15.1 %)	80 (30.5 %)	
	400,000 and over	11 (12.8 %)	105 (40.1 %)	
Work participation	Fully working	13 (15.1 %)	123 (45.6 %)	25.599 (0.000)***
	Partially working	5 (5.8 %)	55 (20.4 %)	9.862 (0.002)**
	Full sick-leave	14 (16.3 %)	73 (27.0 %)	4.088 (0.043)*
	No participation in the labour market	32 (37.2 %)	9 (3.3 %)	73.452 (0.000)***
	School/studies	21 (24.4 %)	9 (3.3 %)	37.578 (0.000)***
	Other	1 (1.2 %)	1 (0.4 %)	0.733 (0.392)
Use of health care services				
Visits to a GP in the past 12 months	None	2 (2.3 %)	2 (0.7 %)	13.241 (0.004)**
	1–2	22 (25.6 %)	35 (13.1 %)	
	3–4	24 (27.9 %)	58 (21.7 %)	
	5+	38 (44.2 %)	172 (64.4 %)	
Visits to hospital (outpatient/inpatient) in the past 3 years	None	41 (47.7 %)	160 (59.7 %)	6.624 (0.085)
	1–2	29 (33.7 %)	80 (29.9 %)	
	3–4	12 (14.0 %)	17 (6.3 %)	
	5+	4 (4.7 %)	11 (4.1 %)	
History of psychiatric treatment	Yes	50 (58.1 %)	134 (49.8 %)	1.809 (0.179)
	Outpatient clinic, <18 years	9 (10.5 %)	11 (4.1 %)	4.983 (0.026)*
	Outpatient clinic, >18 years	23 (26.7 %)	44 (16.4 %)	4.592 (0.032)*
	Private practice	16 (18.6 %)	78 (29.0 %)	3.615 (0.057)
	Hospitalization	12 (14.0 %)	6 (2.2 %)	18.606 (0.000)***
	Other	2 (2.3 %)	12 (4.5 %)	0.784 (0.376)
Present medication (for psychiatric problems)	Yes	40 (48.2 %)	87 (33.0 %)	6.319 (0.012)*
	Antipsychotics	10 (12.0 %)	2 (0.8 %)	24.111 (0.000)***
	Antidepressants	25 (30.1 %)	64 (24.2 %)	1.144 (0.285)
	Anxiolytics	13 (15.7 %)	11 (4.2 %)	12.963 (0.000)***
	Sleeping pills	11 (13.3 %)	23 (8.7 %)	1.473 (0.225)
	Other	7 (8.4 %)	5 (1.9 %)	8.089 (0.004)**

*Sig. *p* <0.05 **Sig. *p* <0.01 ***Sig. *p* <0.001

In the RTW group, most of the patients participated in the work arena (Table 1), and were entitled sick-leave benefits: 93.0 % were either working fully or partly, or were completely on sick-leave, compared with 37.3 % of the patients in the regular outpatient group. On the other hand, a greater percentage of the patients in the regular outpatient clinic than in the RTW clinic reported that they were mainly studying (24.4 % compared with 3.3 %; *p* = 0.000).

As shown in Table 1, the number of visits to a GP in the previous year differed significantly between the two groups (*p* = 0.004). 64.4 % of the patients in the RTW group had visited a GP five or more times in the previous year, compared with 44.2 % of the patients in the regular outpatient group. On the other hand, patients in the RTW group had less experience with child and adolescent psychiatric outpatient clinics (4.1 % compared with 10.5 %; *p* = 0.026), adult psychiatric outpatient clinics (16.4 % compared with 26.7 %; *p* = 0.032) and psychiatric hospitalization (2.2 % compared with 14.0 %; *p* = 0.000). Thirty-three per cent of the patients in the RTW group reported using medications for psychiatric problems, compared with 48.2 % of the patients in the regular outpatient group (*p* = 0.012). Examining different types of medications, the RTW group used significantly fewer antipsychotics (0.8 % compared with 12.0 %; *p* = 0.000), anxiolytics (4.2 % compared with 15.7 %; *p* = 0.000) and other unspecified medications (1.9 % compared with 8.4 %; *p* = 0.004).

### Clinical data

#### Mental health status

As shown in Table 2, patients in the regular outpatient clinic had higher total scores on CORE-OM (*p* = 0.033), including the Problem (*p* = 0.039) and Risk (*p* = 0.000) subscales. Of the four specific categories in the Problem subscale, only Depression was significantly higher (*p* = 0.028) in the regular outpatient clinic. Of the two subcategories in the Risk subscale, Risk to oneself was significantly higher (*p* = 0.000) in the regular outpatient clinic.

**Table 2:** Clinical data

	Regular outpatients *N* = 86		Return to work *N* = 270		Significance testing	
	Mean	SD	Mean	SD	F	Sig.
CORE-OM						
CORE-OM total	18.3	6.49	16.7	5.65	4.57	0.033*
Well-being	23.5	8.10	23.0	7.50	0.27	0.606
Problem	23.2	8.21	21.2	7.33	4.30	0.039*
Anxiety	22.0	9.90	19.9	8.63	3.77	0.053
Depression	24.5	8.55	22.2	8.76	4.85	0.028*
Somatic	22.2	10.64	21.6	9.76	0.23	0.630
Trauma	23.5	10.88	21.7	9.81	2.08	0.150
Function	17.6	6.78	16.8	5.71	1.28	0.259
Relations	16.5	8.32	15.4	7.58	1.22	0.269
General	20.1	7.56	19.2	6.54	1.08	0.299
Social	16.3	9.22	15.7	7.61	0.42	0.516
Risk	6.1	6.31	3.3	4.87	18.25	0.000***
Risk to self	8.1	8.18	4.2	6.67	19.01	0.000***
Risk to others	2.0	5.71	1.3	3.87	1.77	0.184
CORE-OM total without risk	20.8	7.10	19.6	6.22	2.51	0.114
HRQoL						
15D	0.772	0.12	0.785	0.10	1.01	0.317
GAF						
GAF Symptom	56.1	8.16	60.0	7.85	16.66	0.000***
GAF Function	59.3	11.05	62.1	9.71	5.64	0.018*
Diagnoses	*N*	%	*N*	%	Chi-Square	Sig.
Common mental disorders	53	65.4 %	218	93.6 %	40.24	0.000***
Severe psychopathology	5	6.2 %	3	1.3 %	5.78	0.016*
Other mental disorders	23	28.4 %	12	5.2 %	32.79	0.000**

*Sig. *p* <0.05 **Sig. *p* <0.01 ***Sig. *p* <0.001

In analyses not shown in detail, a greater proportion of the regular outpatients had CORE-OM scores in the highest level of severity (above 25), compared to RTW patients (16.7 % vs. 6.1 %; χ^2^ = 9.100; sig. = 0.003). No such differences were found for any of the other levels of severity. Thus, the average differences between the two clinics on the different CORE scales could be due to the greater proportion of patients with especially high scores in the regular clinic. Consequently, further ANOVA analyses were done, leaving out the most severe patients in both clinics. In these results, the mean differences between the two clinics on the total CORE-OM, Problem and Depression scores went insignificant, whereas the differences remained significant for the Risk and Risk to oneself scores.

The two groups did not differ significantly on the HRQoL (15D score). Table 2 shows that patients in the RTW group received significantly higher scores on both the GAF-S (60.0; *p* = 0.000) and the GAF-F (62.1; *p* = 0.018) than did the patients in the regular outpatient clinic (GAF-S 56.1 and GAF-F 59.3). Among the regular outpatient clinic patients, a significantly higher percentage had other psychiatric disorders (28.4 %; *p* = 0.000) and more severe psychopathology (6.2 %; *p* = 0.016) compared with patients in the RTW clinic (5.2 % and 1.3 %, respectively). Psychiatric diagnoses were given to 314 patients, while 42 patients received z-diagnoses (36 with Z00.4 general psychiatric examination, and six with other z-diagnoses). The largest group of patients with Z00.4 only met for psycho-educative courses in the RTW clinic (*n* = 30), and therefore they were never diagnosed. Analyses of the distribution of diagnoses between the two clinics were done with and without the z-diagnoses, and the conclusions were the same. However, because the results without the z-diagnoses were considered to give the most representative picture, they are presented here.

### Logistic regression

Both univariable and multivariable logistic regression analyses were performed, with odds ratios above one indicating higher probability of being treated in the RTW clinic (Table 3). All of the variables that differed significantly between the two groups in the initial analyses (see Tables 1 and 2) also differed in the univariable regression analyses. In the multivariable analysis, the odds of present work participation (OR = 18.25; *p* = 0.000) and being diagnosed with CMD (OR = 2.98; *p* = 0.042) were higher in the RTW clinic. The odds of history of psychiatric hospitalization (OR = 0.06; *p* = 0.043) were lower among RTW patients.

**Table 3:** Logistic regression analyses, univariable and multivariable

	Univariable			Multivariable		
	OR	95 % C.I.	Sig.	OR	95 % C.I.	Sig.
Higher age	1.67	1.26–2.21	0.000***	1.12	0.70–1.79	0.636
Living with partner	2.18	1.30–3.67	0.003**	0.96	0.41–2.28	0.930
Living with children	2.60	1.37–4.96	0.004**	1.28	0.44–3.70	0.650
Present work participation	22.29	11.76–42.25	0.000***	18.25	7.18–46.36	0.000***
Higher income	2.55	1.99–3.28	0.000***	1.47	0.99–2.19	0.054
History of psychiatric hospitalization	0.14	0.05–0.39	0.000***	0.06	0.00–0.92	0.043*
Treated in child and adolescent outpatient clinic	0.37	0.15–0.91	0.031*	0.45	0.10–1.96	0.284
Treated in adult outpatient clinic	0.54	0.30–0.95	0.034*	2.18	0.64–7.43	0.214
Present medication for psychiatric problems	0.53	0.32–0.87	0.013*	0.83	0.33–2.09	0.686
Presently using antipsychotics	0.06	0.01–0.26	0.000***	0.17	0.02–1.19	0.074
Presently using anxiolytics	0.23	0.10–0.55	0.001**	0.38	0.10–1.41	0.146
Severe CORE-OM score	0.32	0.15–0.69	0.004**	0.45	0.12–1.71	0.242
Diagnosed with CMD	7.68	3.83–15.39	0.000***	2.98	1.04–8.52	0.042*
Higher GAF function	1.03	1.00–1.06	0.022*	0.98	0.93–1.03	0.413
Higher GAF symptom	1.06	1.03–1.10	0.001**	1.05	0.98–1.12	0.189

The dependent variable is patient group (regular outpatient or RTW). Odds ratio (OR) for being treated in RTW. OR above one indicating higher probability of being treated in the RTW clinic

*Sig. *p* <0.05 **Sig. *p* <0.01 ***Sig. *p* <0.001

## Discussion

The main finding of this study is that patients in the RTW clinic had lower levels of self-reported psychological distress than patients in the regular outpatient group, but both groups were within moderate levels of severity. Another important finding is that the RTW clinic reaches its intended target group, in that the RTW patients had jobs and were entitled to sick-leave benefits, and most had been diagnosed with CMDs.

### Comparing the two groups

The results showed that patients in the RTW clinic had lower levels of self-reported psychological distress as measured by CORE-OM. However, both groups were within moderate levels of severity. A greater proportion of the regular outpatients had CORE-OM scores in the highest level of severity. No such differences were found for any of the other levels of severity. Thus, the average difference between the two clinics on the total CORE-OM score seems to be due to the greater proportion of patients with especially high scores in the regular clinic. Consequently, it seems that the RTW group did not include the most severely ill patients, but otherwise it overlapped substantially with the regular outpatient group. The large overlap between the two groups raises the question of whether the RTW programme should more accurately be described as an extension of existing services than as a specific programme.

The diagnoses the therapists gave the patients reflected the same pattern. Although the majority of patients in both clinics were diagnosed with CMDs, the majority was much greater in the RTW sample than in the regular outpatient sample. Similarly, few patients were diagnosed with severe psychopathology, but the proportion was significantly greater in the regular outpatient group. The symptom and function GAF scores that the therapists assigned to the patients differed between the two groups, with lower scores for the regular outpatient group. However, although the 3–4-point differences on the GAF scale were statistically significant, and probably clinically relevant, they were not huge.

As expected, regular outpatients were more likely to have had previous mental health care treatment. Somewhat surprisingly, however, half of the patients in the RTW group also had a history of treatment in mental health care. This indicates that the RTW group not only consisted of recent single-episode cases; it also included patients with a longer history of illness. However, when we compared the specific histories of the two groups, we found that the regular outpatient group had a more severe history in secondary health care, and for some patients that history had started earlier in life. Consistent with this was the finding that about half of the patients in the regular outpatient group was using medications for psychiatric problems, whereas only one third in the RTW group did so, which might reflect the slightly higher levels of symptoms and the longer history of treatment for the regular outpatient group. The fact that two thirds of patients in the RTW group had seen their GPs five or more times in the previous year might indicate both attempts to treat a recent mental health problem and visits to evaluate the need for sick-leave. The fact that so many in the RTW group had had fairly close contact with their GPs and yet were still referred for specialized health care could suggest that treatment in primary care was not sufficient for these patients. But this could also be the result of a GP preference for RTW services or that the GP felt these were more appropriate for the patient.

With respect to subjective quality of life, both groups reported reduced levels relative to population studies [43], but there was no difference between the two clinics. It is interesting that the patients in the RTW group experienced the same reduction in subjective quality of life, even though their symptoms were slightly less severe. Perhaps this indicates that being on sick-leave, or at risk of requiring it, is a substantial threat to a person’s quality of life. This would be consistent with other research that shows positive correlations between work participation and both health and psychological well-being [48–50].

Contrary to our expectations, the two groups differed on several socio-demographic variables. Patients in the RTW intervention were somewhat older and more likely to live in relationships, have children and be working, and they also had higher incomes. To some extent, the age difference between the two groups can probably explain the differences in civil status, children and work participation. However, it is also possible that these variables indicate lower functioning in the regular outpatient group over time, more so than the present level of symptoms. The difference in income can be explained by the fact that almost all of the patients in the RTW group participated in the work arena, whereas this was true only for four out of ten in the regular outpatient group. Because participation in work is an inclusion criterion for the RTW programme, we expected the high work participation numbers for the RTW group. The low percentage of patients participating in work in the regular outpatient group is not so obviously explained. About one out of four patients in the outpatient group reported that they were studying, and it is interesting to note that there was no significant difference in education level between the two groups. If the regular outpatient group had a generally lower level of functioning, we would expect this to be associated with a lower education level as well. Perhaps this finding shows that the education system in Norway is fairly inclusive. It is also possible that state-sponsored education is used as an alternative to unemployment or social welfare in Norway.

### Selection to the two different clinics

The multivariable regression analysis indicated that three variables seemed to contribute uniquely to the explanation of which patients were included in the respective clinics: work participation, previous psychiatric hospitalization and current diagnosis. This makes sense, because work participation is an inclusion criterion for the RTW programme, and both current diagnosis and previous psychiatric hospitalization can be seen as indicators of the severity of psychopathology. However, it is interesting that the severity of symptoms did not offer any unique contribution to the explanation of which patients were in the respective clinics. This might reflect a tendency among both the referring GPs and the clinicians responsible for intake to give more weight to the patients’ anamnesis than to their present status when choosing between treatment alternatives. This makes some sense clinically, because the severity of earlier episodes is relevant to assessing such things as the consequences of a refused intake or time on a waiting list.

### Does the RTW clinic reach its intended target group?

Our results showed that a clear majority of patients at the RTW clinic have jobs and are entitled to sick-leave benefits. Even so, a small group did not participate in the work arena when they answered the questionnaire. This can largely be explained by the fact that inclusion in the program is based on information in the referrals from the GPs. If patients are referred towards the end of a long period of sick-leave, they sometimes lose their sick-leave benefits before they actually start treatment. These patients are then still accepted into the program. A large majority of RTW patients have CMDs, and the sample fits the description of “less severe mental disorders”. Thus, we conclude that the clinic reaches its intended target group. Often, patients had already been treated in primary health care settings before being referred to the RTW clinic. This is not a requirement of the programme, but it indicates that specialized health care was thought to be needed. As we have seen, the overlap in mental health status between the two groups was quite large. This raises the question of whether it is appropriate to view the RTW population as a subgroup of the regular outpatient population rather than as a distinct group. It may well be that GPs refer patients to the RTW programme whom they suspect might otherwise not be offered specialized mental health care treatment. This may be because such patients have done well earlier in life: they have a job, a family and less of a history in mental health care, and ordinarily they might be seen as too functional for a regular outpatient clinic.

### Strengths and weaknesses

A strength of this study is that we used well-established instruments, and combined information from both patients, therapists and medical records. This gave us a more complete picture of the patients’ health status. Another strength is that the naturalistic design allows us to claim that the findings have direct relevance to clinical practice, at least more so than do findings from experimental designs with highly selective samples. However, the naturalistic setting also has some weaknesses. Both recruiting the participants and collecting the questionnaires were challenging in this study. Many patients were never asked to participate. In the regular outpatient clinic almost one in five patient were excluded by the therapists. About one third and two fifths of the patients considered for participation in the two clinics respectively, did not return the questionnaire. This means that both self-selection and selective recruitment might have introduced a bias in the final samples. However, another strength of the study is that we were able to collect some data for the whole population. On those variables that could be checked, there were no significant differences between responders and non-responders in either of the clinics. Thus, we conclude that the findings from the samples can be extrapolated to some degree to the whole population, when it comes to age, gender, GAF scores and diagnoses. On other variables, the results must be interpreted with some caution.

## Conclusions

This study showed that, relative to outpatients in specialized mental care, RTW patients had lower levels of self-reported psychological distress as measured by CORE-OM. However, both groups were within moderate levels of severity. There was no difference in reduction in health related quality of life. In addition, patients at the RTW clinic were in the intended target group for the RTW programme. Even so, we can ask whether this group of patients could have been treated just as well in a regular outpatient clinic, as their symptoms and diagnoses to a large extent overlapped with those of the patients in the regular outpatient clinic. About half of the RTW patients had had recurrent episodes of mental health problems (as indicated by previous treatment). Many had been treated in primary care settings before referral. All of this indicates that this group has significant health problems that can profit from treatment in specialized health care settings. This has implications for the ongoing debate in which patients in the RTW programme are assumed to be prioritized over patients who are more in need of treatment because of their severe diagnoses. Perhaps a more relevant discussion would be why the specialized health care does not have the capacity to treat these patients in the first place. The large proportion of CMD diagnoses in this group indicates a good prognosis for these patients, as there are effective treatments for such disorders. It still remains to be seen whether the goal of reducing the cost to society of sick-leave benefits will be achieved. This deserves more study, and we will examine this in a future prospective longitudinal study on this sample, based on questionnaires and register data on sickness absence.

## Abbreviations

CMD, common mental disorder; CORE-OM, clinical outcomes in routine evaluation outcome measure; DSM-IV, diagnostic and statistical manual of mental disorders – fourth edition; EQ-5D, the EuroQOL five-dimension questionnaire; GAF, the global assessment of functioning; GP, general practitioner; HRQoL, health-related quality of life; IAPT, improving access to psychological therapies; ICD-10, the international classification of diseases – tenth edition; OECD, organisation for economic co-operation and development; RTW, return to work; SF-6D, short form 6D; SPSS, statistical package for the social sciences; WHO, World Health Organization
